# The Differences of Population Birth Defects in Epidemiology Analysis between the Rural and Urban Areas of Hunan Province in China, 2014–2018

**DOI:** 10.1155/2021/2732983

**Published:** 2021-04-21

**Authors:** Lili Xiong, Qiongying Chen, Aihua Wang, Fanjuan Kong, Donghua Xie, Zhiqun Xie

**Affiliations:** Hunan Province Maternal and Children Health Care Hospital, Changsha, China

## Abstract

**Objectives:**

To compare the differences of epidemiology analysis in population birth defects (BDs) between the rural and urban areas of Hunan Province in China.

**Methods:**

The data of population-based BDs in Liuyang county (rural) and Shifeng district (urban) in Hunan Province for 2014–2018 were analyzed. BD prevalence rates, percentage change, and annual percentage change (APC) by sex and age were calculated to evaluate time trends. Risk factors associated with BDs were assessed using simple and multiple logistic regression analyses.

**Results:**

The BD prevalence rate per 10,000 perinatal infants (PIs) was 220.54 (95% CI: 211.26-230.13) in Liuyang and 181.14 (95% CI: 161.18-202.87) in Shifeng. Significant decreasing trends in BD prevalence rates were noted in the female PIs (APC = −9.31, *P* = 0.044) and the total BD prevalence rate in Shifeng (APC = −14.14, *P* = 0.039). Risk factors for BDs were as follows: rural area, male PIs, PIs with gestational age < 37 weeks, PIs with birth weight < 2500 g, and migrant pregnancies.

**Conclusions:**

We should focus on rural areas, reduce the prevalence of premature and low birth weight infants, and provide maternal healthcare services for migrant pregnancies for BD prevention from the perspective of population-based BD surveillance.

## 1. Background

Birth defects (BDs)—also called congenital anomalies—are defined by the World Health Organization (WHO) as structural, functional, or biochemical-molecular defects present at birth, whether or not detected at that time [1]. The WHO estimates that every year, 6% of all newborns worldwide are born with serious BDs, with a BD prevalence rate of 4.72% in developed countries, 5.57% in middle-income countries, and 6.42% in low-income countries [2, 3]. In China, the BD rate is reported to be 5.6%, and 900,000 newborns are found to have BDs each year [4]. BD surveillance data indicate that among China's provinces, Hunan ranked third in 2011 in terms of the BD prevalence rate, fourth in 2012, and fifth in 2013.

Different countries have different BD surveillance systems. Hospital surveillance and population surveillance are the two main methods of BD surveillance. The European Network of Congenital Anomaly Registers was established in 1979 and is a high-quality network of population-based congenital anomaly registries across Europe for BD surveillance and research [5, 6]. In the United States, the National BDs Prevention Network publishes state-level data on major structural BDs to advance the field of BD surveillance and epidemiology, with the first Congenital Malformations Surveillance report published in 1997 [7]. Hospital BD surveillance is conducted in continents and countries, such as Latin America, China, and South Korea [4, 8, 9]. BD surveillance systems in China started in 1986. These hospital-based surveillance systems monitored about 3.63 million births, which accounted for about 22% of all births in China [10]. This surveillance method is considered to work well in China. With the growth of economies and the development of healthcare services worldwide, it is necessary to build population-based BD surveillance systems that can supplement hospital-based surveillance systems [4].

Until now, most BD research studies in China used data from hospital-based systems; no studies have used population-based BD surveillance data. This study is aimed at providing an epidemiologic profile of population-based BDs in Liuyang county (rural) and Shifeng district (urban) in Hunan Province using data from the Chinese BDs Population Monitoring Network for 2014–2018. We aimed to understand the occurrence of BDs in rural and urban areas and provide basic data and policy suggestions for the prevention of BDs from the perspective of population-based BDs.

## 2. Material and Methods

### 2.1. Study Population

Hunan Province had selected Liuyang in Changsha city and Shifeng in Zhuzhou city as rural and urban population BD surveillance sites since 2008. The reasons for selection were as follows. (1) The local health administrative department pays attention to and supports the surveillance work, supporting funds and staff. (2) The health service indicators of children and maternal system management rate and hospital delivery rate were more than 80%. (3) Health promotion such as prepregnancy healthcare, prenatal health, prenatal screening, newborn disease screening, and hearing screening had been carried out. (4) The local administration agreed to participate in the program. The monitoring subjects were the perinatal infants (PIs; including stillbirth, dead fetus, or live births) delivered by the mothers living in the monitoring area (including the mothers with local household registration and those with nonlocal household registration living in the monitoring area for more than one year). The monitoring period is from 28 weeks of gestation (if the gestational age is unclear, a birth weight of 1000 grams or more can be referred to) to 42 days after birth, during which time the BD is diagnosed. The two surveillance sites are organized and implemented by provincial administrative departments under the unified implementation plan formulated by the National Maternal and Child Health Monitoring Office.

### 2.2. Data Collection and Data Source

The maternal and child healthcare workers at the community health service centers in urban areas and village doctors in rural areas are responsible for collecting in the regional jurisdiction information related to all PIs delivered after the 28 weeks of gestation and filling in the “birth status and infant follow-up registration form,” which included information on family conditions, infant conditions, BD diagnosis conditions, and results of PI follow-up. They would follow up the live infants until 42 days after birth by way of postpartum visits and record the information on follow-up results on the form. The “birth status and infant follow-up registration form” of all the infants and “registration form of BDs” of those diagnosed with BDs were entered into the Chinese Birth Defects Population Monitoring system.

The diagnosis of BDs was based on the Chinese National Criteria of Birth Defects and Tiny Deformities and the clinical modification codes as congenital malformations, deformations, and chromosomal abnormalities (codes Q00–Q99) of “International Statistical Classification of Diseases and Related Health Problems, Tenth Revision (ICD-10)”. The 24 types of BDs monitored were categorized by the system affected, including neurologic system, craniofacial system, gastrointestinal system, urogenital system, musculoskeletal system, cardiovascular system, respiratory system, genetic metabolic diseases, genetic syndrome, and other BDs (i.e., those BDs do not belong to the abovementioned 9 systems). The 24 types of BDs listed in our previous research were categorized by the clinical and physical signs, including morphological and structural abnormalities, functional and metabolic abnormalities, and mental-behavioral abnormalities [11].

Cases of BDs should be diagnosed by district, county, or above medical institutions and confirmed by the expert groups for the monitoring of population BDs established by the monitoring district and county. Those pregnant women with defective fetuses found in prenatal diagnosis who want to terminate pregnancies must go to medical institutions qualified for prenatal diagnosis, which had the higher ability on the diagnostic level of B-ultra, medical genetics, obstetrics, pediatrics, pathology, or clinical laboratory. The diagnostic information about BDs would be filled in the registration forms for BDs including name, position, diagnosis time, and diagnosis based on BD diagnosis conditions.

### 2.3. Quality Control and Other Issues

**Figure 1:**
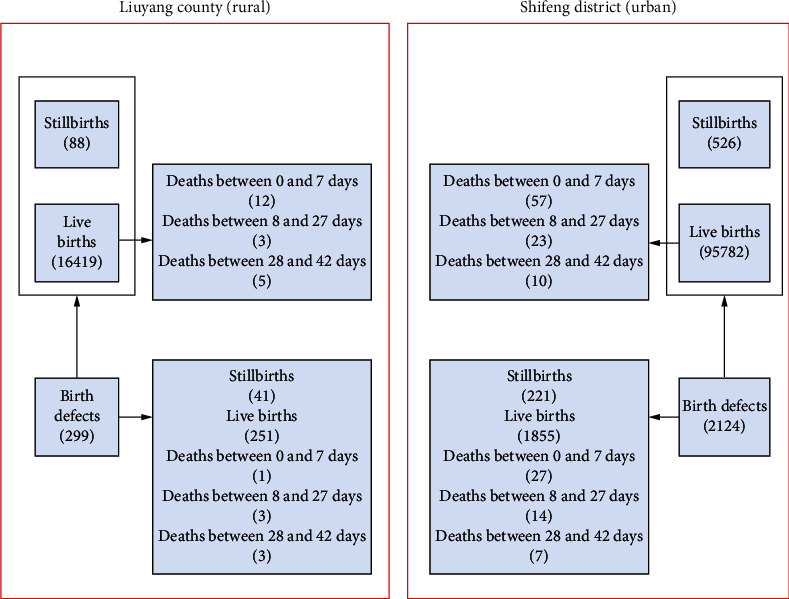
The flow chart showing how the study sample was selected from Liuyang county (rural) and Shifeng district (urban), Hunan Province, China, from 2014 to 2018.

**Figure 2:**
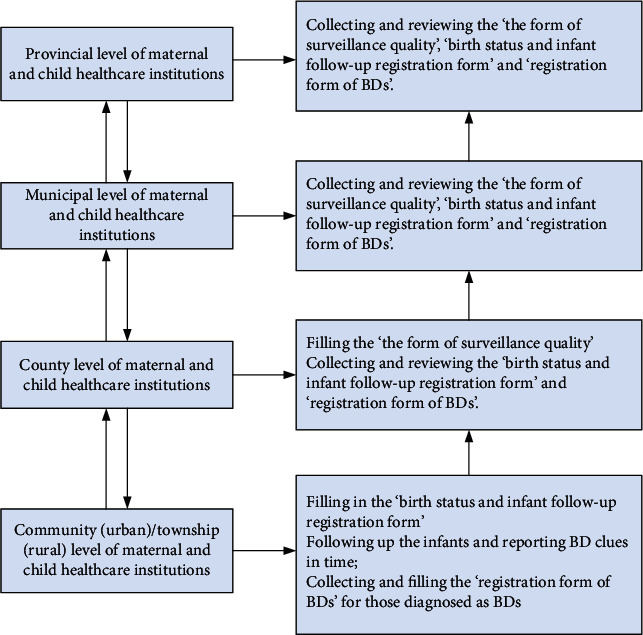
The process of the data collection and quality control in Liuyang county (rural) and Shifeng district (urban), Hunan Province, China, from 2014 to 2018.

The surveillance staff at the county level of maternal and child healthcare institutions filled in “the form of surveillance quality” including numbers of PIs, perinatal deaths, and BDs every quarter. Then, the form was returned to the surveillance staff at the municipal level of maternal and child healthcare institutions, together with the requirement of the underreporting rate of major BDs no more than 1%, the number of unreported births no more than 1%, the completeness of the report forms more than 99%, errors on report forms no more than 1%, and errors resulted from data entry no more than 1%. The provincial level of maternal and child healthcare institutions carried out the same review procedure when the municipal level had passed the review. The flow chart of population BD monitoring is listed in Figure 1. A flow chart showing how the data of the sample was selected in Liuyang and Shifeng from 2014 to 2018 is presented in Figure 2. Overall, 2423 BDs were identified. The total number of PIs in this study was 112,815. All data except for the name, address, and other information identifying the study cases in the study were regularly uploaded from the Chinese BDs Population Monitoring Network to conduct statistical analysis only. This study was conducted in compliance with local and national regulations and was approved by the Ethics Review Committee of Hunan Province Maternal and Children Health Care Hospital. The requirement for obtaining informed consent was waived because of the retrospective design of this study and only usage of monitoring data that could not identify the subjects. This study was carried out in accordance with the principles of the Declaration of Helsinki. The prevalence of BDs was expressed per 10,000 PIs.

### 2.4. Statistical Analysis

The total prevalence and 95% confidence intervals (CI) of BD prevalence rates grouped by maternal age and infants' sex and 95% CI were calculated for five 1-year time intervals from 2014 to 2018. A regression line was fitted to the natural logarithm of the rates weighted by the number of cases to look specifically at time trends; that is, *y* = *α* + *βx* + *ε*, where *y* = ln(rate) and *x* = calendar year. The APC and 95% CI for the BD prevalence grouped by maternal age and infants' sex were calculated based on the Joinpoint regression modeling using Joinpoint software, version 4.5 (US National Cancer Institute), to quantify the time trends. Line charts were constructed to graphically display the trends in BD prevalence rates by sex and maternal age groups. The distributions of epidemiological and delivery characteristics were compared between the BD group and the non-BD group using simple logistic regression. The dependent variable of logistic regression analysis was whether a perinatal infant had BDs (no/yes). Multivariable logistic regression analysis was used to calculate aORs (adjusted odds ratios) and 95% CI. Only variables identified as significantly associated with the dependent variable at *P* < 0.05 in the simple logistic regression analyses were included in the model. All analyses were conducted using SPSS version 22 (IBM Corp., Armonk, NY, USA). All statistical tests were two-sided, and *P* values less than 0.05 were considered statistically significant.

## 3. Results

Table 1 presents the number of PIs, BDs, and BD prevalence rates per 10,000 PIs (95% CI) by sex for the two study areas for the 5-year observational period. We observed 2423 BDs among 112,814 PIs. There were 2124 BDs among 96,307 PIs, and the BD prevalence (95% CI) was 220.54 (95% CI: 211.26-230.13) in Liuyang. There were 2124 BDs among 96,307 PIs in Liuyang, and there were 299 BDs among 16,507 PIs in Shifeng. The BD prevalence (95% CI) was 181.14 (95% CI: 161.18-202.87) in Shifeng. The prevalence among male PIs was higher than that among female PIs.

**Figure 3:**
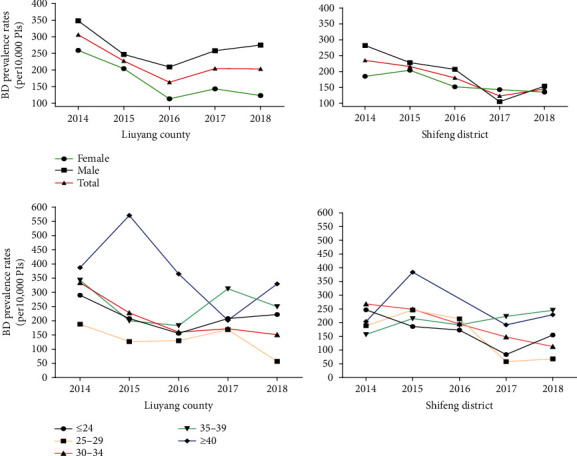
The prevalence of BDs by sex and maternal age groups in Liuyang county (rural) and Shifeng district (urban), Hunan Province, China, from 2014 to 2018.

Table 2 presents the number of PIs, BDs, and BD prevalence rates in the study areas by maternal age groups. Except for the mothers aged <40 years, the prevalence of the other age groups in Liuyang was higher than that in Shifeng. Mothers aged ≥40 years in Liuyang (352.82 per 10,000 PIs, 95% CI: 273.43-448.07) and 35–39 years in Shifeng (212.37 per 10,000 PIs, 95% CI: 147.07-296.76) had the highest prevalence of BDs among the maternal age groups. The time trends of BD prevalence rates by sex and maternal age groups in Liuyang and Shifeng from 2014 to 2018 are depicted in Figure 3. Although the overall prevalence rates in Liuyang decreased over the study period from 306.49 to 203.39 per 10,000 PIs, the annual percentage change (APC) did not show statistical significance (APC = −8.83, *P* = 0.243) (Table 3). However, we observed significant decreasing BD trends in the following: mothers aged 30–34 years in Liuyang (APC = −17.03, *P* = 0.037), female PIs in Shifeng (APC = −9.31, *P* = 0.044), mothers aged 30–34 years in Shifeng (APC = −20.06, *P* = 0.002), and the total time trend for Shifeng (APC = −14.14, *P* = 0.039).

Detailed maternal epidemiological and delivery characteristics related to PIs with BDs and non-BDs, BD prevalence rates, and odds ratios (ORs) are presented in Table 4. The average BD prevalence rate in Liuyang was significantly higher than that in Shifeng (OR = 1.223, 95% CI: 1.082–1.382). The average BD prevalence rate was significantly higher in male PIs than in female PIs (OR = 1.536, 95% CI: 1.413–1.669). The average BD prevalence in mothers aged 35–39 years and ≥40 years was significantly higher than that in mothers aged 25–29 years (OR = 1.206, 95% CI: 1.1046–1.389 and OR = 1.572, 95% CI: 1.238–1.998, respectively). The average BD prevalence in PIs at <37 gestational weeks was significantly higher than that in PIs at 37–42 gestational weeks (OR = 5.392, 95% CI: 4.855–5.990). The average BD prevalence rate in PIs with a birth weight < 2500 g was significantly higher than that in PIs weighing 2500–4000 g (OR = 5.878, 95% CI: 5.273–6.553). The average BD prevalence in twin PIs was significantly higher than that in singleton PIs (OR = 1.540, 95% CI: 1.237–1.916). Finally, the average BD prevalence in PIs whose mothers have nonlocal registration but residence with <1 year (OR = 3.052, 95% CI: 2.105–4.426) and ≥1 year (OR = 3.779, 95% CI: 2.824–5.055) was significantly higher than that in PIs whose mothers have local registration.

Factors associated with BDs with the dependent variable of BD occurrence (yes/no) in the multiple logistic regression model are shown in Table 5. Risk factors for BDs were as follows: rural areas compared with urban areas (adjusted OR (aOR) = 1.366, 95% CI: 1.203–1.551), male PIs compared with female PIs (aOR = 1.564, 95% CI: 1.437–1.702), <37-gestational week PIs compared with 37–42-week PIs (aOR = 2.589, 95% CI: 2.211–3.033), <2500 g PIs compared with 2500–4000 g PIs (aOR = 3.417, 95% CI: 2.899–4.027), mothers with nonlocal certificate registries but residence with 1 year and ≥1 year compared with local certificate registries (aOR = 3.349, 95% CI: 2.284–4.912 and aOR = 4.197, 95% CI: 3.103–5.676, respectively), and mothers aged ≥40 years compared with mothers aged 25–29 years (aOR = 1.317, 95% CI: 1.032–1.682).

## 4. Discussion

This was the first study to describe the epidemiology of BDs based on population surveillance data from Hunan Province in China. The total BD prevalence rates in Liuyang and Shifeng from 2014 to 2018 were 220.54 and 181.14 per 10,000 PIs, respectively. These rates differed from those based on hospital surveillance data reported by our previous study that found the total prevalence rates of BDs in rural and urban areas of Hunan Province (2005–2014) to be 175.41 and 209.66 per 10,000 PIs, respectively [11]. Although Liuyang county and Shifeng district were chosen as representatives of rural and urban population monitoring areas in Hunan Province, some studies with larger simples had shown similar results to our findings based on the population study [12]. The prevalence rates of BDs were inconsistent with the conclusions of other studies in Dalian city and Henan Province of China and nationwide based on hospital surveillance data that found higher BD prevalence rates in urban areas than in rural areas [12–14]. Hospital BD surveillance can ensure the accuracy of malformation diagnosis or the timeliness of obtaining information, especially the surveillance of stillbirth, while population BD surveillance with a long follow-up time covers a wide range of population including the out-of-hospital delivery cases and excluding the migrant pregnancies. Considering that the results of population BD surveillance could obtain more reliable prevalence estimates of BDs, the breadth and depth of information collected at a population level by population BD surveillance should serve as an important data source to guide public health action with the economic development [4, 15].

Greater decreases of prevalence rates were observed in the urban area (62.22%) than in the rural area (50.69%), and only prevalence rates in Shifeng showed a downward trend. The differences in these trends and rural-urban disparities in BD prevalence rates may be explained by rural women having a higher threshold for delaying consultations for a BD infant and tending to seek medical help in urban hospitals with higher diagnostic capabilities [16]. What is more, prenatal diagnosis of BDs from prenatal ultrasound, 3D ultrasound, and ultrafast magnetic resonance imaging has been widely used in urban areas in recent years, and this has increased the sensitivity of prenatal diagnosis of major or minor structural anomalies. It will definitely observe the phenomenon of the prevalence rates of BDs in the rural areas higher than that in the urban areas under the termination of pregnancies with severe congenital structural anomalies after prenatal diagnosis.

We found that in the two study areas, the total prevalence rates of BDs among male PIs (266.65 and 196.68 per 10,000 PIs) were higher than those among female PIs (169.21 and 164.67 per 10,000 PIs). Similarly, other studies reported that more male infants had BDs than female infants [14, 17]. Interactions between sex hormones and organ development might be possible causes of the sex differences found for some congenital anomalies [18]. Our study showed the highest BD prevalence rates among PIs of mothers aged ≥40 years in Liuyang and 35–39 years in Shifeng. Older maternal age is strongly associated with chromosomal BDs such as trisomies 13, 18, and 21 and nonchromosomal BDs [19].

The risk factors for BDs identified in this study were rural residents, male PIs, maternal age ≥ 40 years, multiple births, preterm births, low birth weight, nonlocal pregnancies, and delivery in hospitals. In our study, the rate of BDs in multiple births was 32.27 per 10,000 PIs versus 21.20 per 10,000 PIs in singletons. A study in China's Zhejiang Province found the rate of BDs in multiple births to be 444.16 per 10,000 births versus 266.97 per 10,000 births in singletons using Zhejiang hospital-based BD surveillance system data for 2007–2009 [20]. The mechanisms by which multiple births increase the risk for some BDs remain unclear [21]. This phenomenon may be partly explained by delayed childbirth and consequent infertility, along with increasingly accessible and available assisted reproduction, which resulted in a rise of multiple births and a consequent increase in BDs.

This study showed that BDs were more than twice as common among preterm births (28–36 gestational weeks) and three times as common among retarded births (>42 weeks) compared with term births. Infants born preterm are considered more likely to have major BDs than term infants [22, 23]. The causes of most BDs and the mechanisms or reasons of these BDs that may contribute to preterm birth remain unknown, so prevention and research of BDs and preterm birth had been recognized as an integrated approach to carry out. We found that the prevalence of BDs in migrant pregnancies was higher than that in local pregnancies [24]. Migrant pregnancies may not have benefitted from this series of government policies and healthcare that extends from pregnancy planning to delivery. Therefore, it remains a complex task to provide BD prevention and healthcare for migrant pregnancies. This study also showed that hospital childbirth was a risk factor for BDs compared with delivery in other places. There may be some reasons for this phenomenon. First, the delivery rate in hospitals increased from 58.7% in 1996 to 99.7% in 2015 [25]. Therefore, most infants were delivered in hospitals. Second, pregnancies for which tests during pregnancy showed normal results might have chosen to deliver at home. Third, most infants with BDs were diagnosed in hospitals.

This study illustrated the epidemiological characteristics of BDs in two selected areas of Hunan Province from 2014 to 2018. Although population-based BD surveillance can effectively avoid selection bias, has a longer monitoring time, can collect more BDs diagnosed after delivery, and has a better ability to monitor genetic metabolic diseases and delayed-onset diseases, there were some limitations. First, we could not analyze factors such as smoking, alcohol consumption, socioeconomic status, medical history, prenatal care, or paternal details that may affect BDs because of the limited data collected. Second, this surveillance of BDs only considered 24 of the 110 most frequent and lethal anomalies. Therefore, some BDs might not be detected within a few days after birth, which could lead to the prevalence of BDs in this study being lower than the true prevalence. Third, population BD surveillance based on Liuyang and Shifeng cannot fully represent the whole Hunan Province. However, our report provided an epidemiological analysis of two areas in Hunan Province over 5 years, which provides information that has been heretofore limited in developing countries because of the lack of population BD surveillance. What is more, this study provided pieces of advice for preventing BD occurrence.

## 5. Conclusions

In summary, the prevalence of BDs in population surveillance was different from that in hospital surveillance. The risk factors for BDs identified in this study were rural residents, male PIs, maternal age ≥ 40 years, multiple births, preterm births, low birth weight, nonlocal pregnancies, and delivery in hospitals. Population-based BD surveillance should serve as an important data source to guide local public health action for the prevention and control of BDs.

## Figures and Tables

**(a):** 

Rural	Female-Liuyang county			Male-Liuyang county			Total-Liuyang county		
Year	PIs (*N*)	BDs (*N*)	Prevalence rates (per 10,000 PIs) (95% CI)	PIs (*N*)	BDs (*N*)	Prevalence rates (per 10,000 PIs) (95% CI)	PIs (*N*)	BDs (*N*)	Prevalence rates (per 10,000 PIs) (95% CI)
2014	9126	237	259.7 (227.68-294.95)	10,124	353	348.68 (313.25-387.02)	19,250	590	306.49 (282.26-332.25)
2015	9235	189	204.66 (176.52-236.00)	10,169	252	247.81 (218.16-280.37)	19,404	441	227.27 (206.55-249.51)
2016	9928	113	113.82 (93.80-136.84)	10,996	230	209.17 (183.00-238.02)	20,924	343	163.93 (147.04-182.23)
2017	9474	136	143.55 (120.44-169.80)	10,735	278	258.97 (229.42-291.27)	20,209	414	204.86 (185.60-225.58)
2018	7803	96	123.03 (99.65-150.24)	8717	240	275.32 (241.59-312.45)	16,520	336	203.39 (182.22-226.34)
Total	45,566	771	169.21 (157.47-181.58)	50,741	1353	266.65 (252.63-281.24)	96,307	2124	220.54 (211.26-230.13)

**(b):** 

Urban	Female-Shifeng district			Male-Shifeng district			Total-Shifeng district		
Year	PIs (*N*)	BDs (*N*)	Prevalence rates (per 10,000 PIs) (95% CI)	PIs (*N*)	BDs (*N*)	Prevalence rates (per 10,000 PIs) (95% CI)	PIs (*N*)	BDs (*N*)	Prevalence rates (per 10,000 PIs) (95% CI)
2014	1669	31	185.74 (126.20-263.64)	1807	51	282.24 (210.14-371.09)	3476	82	235.90 (187.62-292.82)
2015	1568	32	204.08 (139.59-288.10)	1665	38	228.23 (161.51-313.26)	3233	70	216.52 (168.79-273.56)
2016	1636	25	152.81 (98.89-225.58)	1737	36	207.25 (145.16-286.93)	3373	61	180.85 (138.33-232.31)
2017	1670	24	143.71 (92.08-213.83)	1798	19	105.67 (63.62-165.02)	3468	43	123.99 (89.73-167.01)
2018	1473	20	135.78 (82.94-209.70)	1484	23	154.99 (98.25-232.56)	2957	43	145.42 (105.24-195.88)
Total	8016	132	164.67 (137.78-195.28)	8491	167	196.68 (167.98-228.87)	16,507	299	181.14 (161.18-202.87)

^a^PIs: perinatal infants; BDs: birth defects; CI: confidence intervals. Population birth defect prevalence rates expressed per 10,000 PIs.

**(a):** 

Rural	≤24-Liuyang county			25-29-Liuyang county			30-34-Liuyang county			35-39-Liuyang county			≥40-Liuyang county		
Year	PIs (*N*)	BDs (*N*)	95% CI prevalence rates (per 10,000 PIs)	PIs (*N*)	BDs (*N*)	95% CI prevalence rates (per 10,000 PIs)	PIs (*N*)	BDs (*N*)	95% CI prevalence rates (per 10,000 PIs)	PIs (*N*)	BDs (*N*)	95% CI prevalence rates (per 10,000 PIs)	PIs (*N*)	BDs(*N*)	95% CI prevalence rates (per 10,000 PIs)
2014	5791	109	188.22 (154.55-227.05)	8744	254	290.48 (255.86-328.49)	3408	114	334.51 (275.93-401.85)	1049	36	343.18 (240.36-475.11)	258	10	387.60 (185.87-712.80)
2015	4403	56	127.19 (96.07-165.16)	9449	197	208.49 (180.39-239.72)	3990	91	228.07 (183.63-280.02)	1247	25	200.48 (129.74-295.95)	315	18	571.43 (338.66-903.10)
2016	3303	43	130.18 (94.22-175.36)	10,281	161	156.60 (133.34-182.74)	5298	85	160.44 (128.15-198.38)	1632	30	183.82 (124.03-262.42)	410	15	365.85 (204.77-603.42)
2017	2829	48	169.67 (125.10-224.96)	8881	185	208.31 (179.37-240.59)	5805	100	172.27 (140.16-209.52)	2201	69	313.49 (243.92-396.75)	493	10	202.84 (97.27-373.03)
2018	2442	14	57.33 (31.34-96.19)	6836	152	222.35 (188.41-260.64)	5023	76	151.30 (119.21-189.38)	1796	45	250.56 (182.76-335.26)	423	14	330.97 (180.94-555.31)
Total	18,768	270	143.86 (127.21-162.09)	44,191	949	214.75 (201.30-228.86)	23,524	466	198.10 (180.52-216.92)	7925	205	258.68 (224.48-296.61)	1899	67	352.82 (273.43-448.07)

**(b):** 

Urban	≤24-Shifeng district			25-29-Shifeng district			30-34-Shifeng district			35-39-Shifeng district			≥40-Shifeng district		
Year	PIs (*N*)	BDs (*N*)	Prevalence rates (per 10,000 PIs) (95% CI)	PIs (*N*)	BDs (*N*)	Prevalence rates (per 10,000 PIs) (95% CI)	PIs (*N*)	BDs (*N*)	Prevalence rates (per 10,000 PIs) (95% CI)	PIs (*N*)	BDs (*N*)	Prevalence rates (per 10,000 PIs) (95% CI)	PIs (*N*)	BDs (*N*)	Prevalence rates (per 10,000 PIs) (95% CI)
2014	634	12	189.27 (97.80-330.62)	1819	45	247.39 (180.45-331.03)	783	21	268.20 (166.02-409.97)	191	3	157.07 (32.39-459.02)	49	1	204.08 (5.17-1137.07)
2015	447	11	246.09 (122.84-440.31)	1659	31	186.86 (126.96-265.23)	843	21	249.11 (154.20-380.79)	232	5	215.52 (69.98-502.95)	52	2	384.62 (46.58-1389.36)
2016	327	7	214.07 (86.07-441.06)	1672	29	173.44 (116.15-249.10)	923	18	195.02 (115.58-308.21)	364	7	192.31 (77.32-396.23)	87	0	0.00 (0.00-424.01)
2017	341	2	58.65 (7.10-211.87)	1428	12	84.03 (43.42-146.79)	1148	17	148.08 (86.26-237.10)	447	10	223.71 (107.28-411.42)	104	2	192.31 (23.29-694.68)
2018	292	2	68.49 (8.29-247.42)	1154	18	155.98 (92.44-246.51)	1057	12	113.53 (58.66-198.31)	367	9	245.23 (112.14-465.53)	87	2	229.89 (27.84-830.42)
Total	2041	34	166.59 (115.37-232.79)	7732	135	174.60 (146.39-206.66)	4754	89	187.21 (150.35-230.38)	1601	34	212.37 (147.07-296.76)	379	7	184.70 (74.26-380.55)

^a^PIs: perinatal infants; BDs: birth defects; CI: confidence intervals. Population birth defect prevalence rates expressed per 10,000 PIs.

**Table 3:** The trends of BD prevalence rates grouped by sex and maternal age groups in Liuyang county (rural) and Shifeng district (urban), Hunan Province, China, from 2014 to 2018.

Gender and age groups among areas	2014		2018		PC (%)	APC (%)	*P* value	95% CI	
	BDs (*N*)	Prevalence rates	BDs (*N*)	Prevalence rates				Limit	Up
Liuyang-female	237	259.70	96	123.03	-111.09	-16.88	0.079	-33.63	4.10
Liuyang-male	353	348.68	240	275.32	-26.65	-4.19	0.546	-21.62	17.11
Liuyang-total	590	306.49	336	203.39	-50.69	-8.83	0.243	-25.58	11.70
Liuyang-≤24 years	109	188.22	14	57.33	-228.31	-18.86	0.181	-44.66	18.98
Liuyang-25-29 years	254	290.48	152	222.35	-30.64	-5.21	0.522	-25.14	20.02
Liuyang-30-34 years	114	334.51	76	151.30	-121.09	-17.03	0.037	-29.74	-2.03
Liuyang-35-39 years	36	343.18	45	250.56	-36.97	-1.80	0.865	-28.24	34.38
Liuyang-≥40 years	10	387.60	14	330.97	-17.11	-12.64	0.311	-38.66	24.42
Shifeng-female	31	185.74	20	135.78	-36.79	-9.31	0.044	-17.33	-0.51
Shifeng-male	51	282.24	23	154.99	-82.10	-17.87	0.091	-36.36	5.99
Shifeng-total	82	235.90	43	145.42	-62.22	-14.14	0.039	-25.17	-1.49
Shifeng-≤24 years	12	189.27	2	68.49	-176.35	-29.30	0.098	-55.55	12.46
Shifeng-25-29 years	45	247.39	18	155.98	-58.60	-15.82	0.204	-39.99	18.10
Shifeng-30-34 years	21	268.20	12	113.53	-136.24	-20.06	0.002	-25.67	-14.03
Shifeng-35-39 years	3	157.07	9	245.23	35.95	9.73	0.063	-0.95	21.55
Shifeng-≥40 years	1	204.08	2	229.89	11.23	-4.45	0.737	-42.49	58.75

^a^PIs: perinatal infants; BDs: birth defects; APC: annual percentage change; CI: confidence interval; PC: percentage change. Population birth defect prevalence expressed per 10,000 PIs. PC and APC between 2014 and 2018 calculated by population birth defect prevalence rates.

**Table 4:** Distribution of epidemiological and delivery characteristics related to BDs and non-BDs, in Liuyang county (rural) and Shifeng district (urban), Hunan Province, China, from 2014 to 2018.

Factors	Classification	Non-BDs (*N*)	BDs (*N*)	Prevalence rates of BDs (per 10,000 PIs) (95% CI)	OR (95% CI)	*P*
Area	Shifeng district	16,228	299	18.092 (16.059-20.124)	Ref	
	Liuyang county	94,274	2124	22.034 (21.107-22.960)	1.223 (1.082-1.382)	0.001
Ethnicity	Han	110,012	2406	21.402 (20.556-22.248)	Ref	
	The others	490	17	33.531 (17.808-49.253)	1.586 (0.976-2.577)	0.062
Age groups	25-29 years	50,884	1084	20.859 (19.630-22.088)	Ref	<0.001
	≤24 years	20,353	471	22.618 (20.599-24.638)	1.086 (0.974-1.212)	0.138
	30-34 years	27,751	555	19.607 (17.992-21.222)	0.939 (0.847-1.041)	0.231
	35-39 years	9305	239	25.042 (21.907-28.177)	1.206 (1.046-1.389)	0.010
	≥40 years	2209	74	32.413 (25.144-39.683)	1.572 (1.238-1.998)	<0.001
Pregnancy times	One time	38,273	846	21.626 (20.185-23.068)	Ref	0.209
	Two times	46,424	980	20.673 (19.392-21.954)	0.955 (0.870-1.048)	0.332
	Three times	25,799	597	22.617 (20.823-24.411)	1.047 (0.942-1.164)	0.397
Parity	One time	46,650	1069	22.402 (21.074-23.730)	Ref	0.027
	Two times	59,525	1244	20.471 (19.345-21.597)	0.912 (0.840-0.991)	0.029
	Three times	4321	110	24.825 (20.242-29.408)	1.111 (0.911-1.355)	0.300
Gender	Female	52,719	903	16.840 (15.751-17.929)	Ref	
	Male	57,783	1520	25.631 (24.359-26.903)	1.536 (1.413-1.669)	<0.001
Gestational weeks	37-42 weeks	105,773	1954	18.138 (17.342-18.935)	Ref	<0.001
	≤36 weeks	4698	468	90.592 (82.763-98.422)	5.392 (4.855-5.990)	<0.001
	≥43 weeks	23	1	41.667 (-44.527-127.861)	2.354 (0.318-17.436)	0.420
Weight of birth	2500-4000 g	100,217	1865	18.270 (17.448-19.091)	Ref	<0.001
	<2500 g	4013	439	98.607 (89.846-107.368)	5.878 (5.273-6.553)	<0.001
	≥4000 g	6266	115	18.022 (14.757-21.287)	0.986 (0.816-1.193)	0.886
Births	Singletons	107,923	2337	21.195 (20.345-22.046)	Ref	
	Twins	2579	86	32.270 (25.557-38.984)	1.540 (1.237-1.916)	<0.001
Place of birth	Hospital	88,186	2111	23.378 (22.393-24.364)	Ref	
	Others	22,316	312	13.788 (12.269-15.308)	0.584 (0.518-0.658)	<0.001
Place of domicile	Local place	109,425	2343	20.963 (20.123-21.803)	Ref	<0.001
	Nonlocal certificate registries but residence with 1 year	459	30	61.350 (40.006-82.694)	3.052 (2.105-4.426)	<0.001
	Nonlocal certificate registries but residence with ≥1 year	618	50	74.850 (54.844-94.857)	3.779 (2.824-5.055)	<0.001

^a^PIs: perinatal infants; BDs: birth defects; OR: odds ratio; CI: confidence interval; Ref: reference. Population birth defects prevalence expressed per 10,000 PIs.

**Table 5:** Factors associated with BD occurrence in the multiple logistic regression model.

Factors associated with BD occurrence	*β*	S.E	Wals	Df	*P*	aOR	aOR (95% CI)	
							Limit	Up
Area of city (Ref: Shifeng district)	-0.31	0.07	23.12	1	<0.001	1.37	1.20	1.55
Gender of male (Ref: female)	0.45	0.04	107.96	1	<0.001	1.56	1.44	1.70
Pregnancy weeks (Ref: 37-42weeks)			140.06	2	<0.001			
≤36 weeks	0.95	0.08	138.94	1	<0.001	2.59	2.21	3.03
≥43 weeks	1.11	1.02	1.18	1	0.277	3.05	0.41	22.68
Weight of birth (Ref: 2500-4000 g)			215.77	2	<0.001			
<2500 g	1.23	0.08	214.78	1	<0.001	3.42	2.90	4.03
≥4000 g	-0.05	0.10	0.28	1	0.598	0.95	0.79	1.15
Multiple births (Ref: singletons)	-0.78	0.12	41.49	1	<0.001	0.46	0.363	0.58
Birthplace of others (Ref: hospital)	-0.45	0.06	50.89	1	<0.001	0.64	0.57	0.72
Mother place of domicile (Ref: local place)			121.13	2	<0.001			
Nonlocal certificate registries but residence with 1 year	1.21	0.20	38.27	1	<0.001	3.35	2.28	4.91
Nonlocal certificate registries but residence with ≥1 year	1.43	0.15	86.68	1	<0.001	4.20	3.10	5.68
Maternal age groups (Ref: 25-29 years)			12.00	4	0.017			
≤24 years	0.05	0.06	0.69	1	0.408	1.05	0.94	1.17
30-34 years	-0.09	0.05	2.78	1	0.095	0.92	0.82	1.02
35-39 years	0.09	0.07	1.36	1	0.243	1.09	0.94	1.26
≥40 years	0.28	0.13	4.89	1	0.027	1.32	1.03	1.68

^a^S.E: standard error; aOR: adjusted odds ratio; CI: confidence interval; Ref: reference.

## Data Availability

The datasets used to support the findings of this study are included within the article.

## Abbreviations

BDs: Birth defects
PIs: Perinatal infants
APC: Annual percentage change
WHO: World Health Organization
CI: Confidence intervals
aORs: Adjusted odds ratios.
